# Digital-droplet PCR assays for IDH, DNMT3A and driver mutations to monitor after allogeneic stem cell transplantation minimal residual disease of myelofibrosis

**DOI:** 10.1038/s41409-022-01566-0

**Published:** 2022-01-19

**Authors:** Daniele Mannina, Anita Badbaran, Christine Wolschke, Evgeny Klyuchnikov, Maximilian Christopeit, Boris Fehse, Nicolaus Kröger

**Affiliations:** 1 University Medical Center Hamburg-Eppendorf, Hamburg, Germany; 2 IRCCS Humanitas Research Hospital, Milano, Italy

**Keywords:** Genetics research, Myeloproliferative disease

**Fig. 1:**
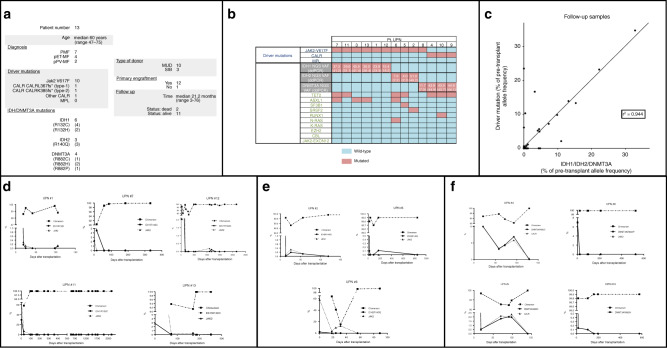
Molecular profiling and post-transplant follow-up. **a** Table: Patients characteristics. **b** Results of molecular profiling of the enrolled patients with myelofibrosis by next-generation sequencing. **c** Correlation of driver mutation and new marker (IDH1/IDH2/DNMT3A) in the follow-up samples. The data are normalized according to the pre-transplant allele frequency (% of the pre-transplant mutation frequency). **d**–**f** the graphs show the molecular follow-up of patients harboring respectively IDH1, IDH2, DNMT3A mutation; different lines and dots display the quantification of JAK2/CALR, IDH/DNMT3A and chimerism.

Primary myelofibrosis (PMF), post-essential thrombocytemia and post-polycytemia vera myelofibrosis (pET/pPV-MF) are potentially curable with allogeneic stem cell transplantation (allo-SCT). Post-transplant relapse is still a major issue, occurring in a widely variable proportion of patients (22–48%) [1, 2]. Although relapse is still unpredictable, relapse probability is influenced by the persistence of minimal residual disease (MRD) after allo-SCT [3]. In addition, timely detection of molecular relapse allows successful treatment with immunotherapeutic strategies [4]. PMF and pET/pPV-MF are characterized by driver mutations involving the genes *JAK2, CALR*, or *MPL* in about 90% of cases [5], and a variable number of non-driver mutations involving epigenetic regulators, histone modifiers, or splicing regulators [6]. The driver mutations are reliable markers of MRD in the allogeneic setting [7–11]. About 10% of all patients with MF (and a higher proportion of transplanted MF patients) [12] harbor none of the driver mutations (triple-negative PMF or pET-MF). Mutations in *IDH* genes occur in 4–9% of MF patients [13–15], and mutations in DNMT3A have been found in 5–10% of patients [16]. We aimed at evaluating the reliability of digital-droplet PCR (ddPCR) assays for quantification of IDH1, IDH2 and DNMT3A mutations as MRD marker for transplanted MF patients. We screened 162 MF patients who underwent allo-SCT between 2013 and 2018 at the Department of Stem Cell Transplantation of the University Medical Center Hamburg-Eppendorf. We performed next-generation sequencing analysis on peripheral blood sample with a customized panel consisting of the following genes: *DNMT3A, IDH1, IDH2, RUNX1, N-RAS, K-RAS, MPL, ASXL1, EZH2, TET2, JAK2 (exons 12 and 14), CBL, SF3B1, SRSF2, CALR*, using Personalized Genome Machine (PGM™; Ion Torrent – Life Technologies/Thermo Fisher). Changes in nucleic acid sequence were annotated using the IGV-Data bank as well as the Ion Reporter software (Life Technologies GmbH/Thermo Fisher). Genetic alterations known to be SNPs were excluded. Among the 162 screened patients, 13 harbor mutations on the *IDH* and *DNMT3A* genes: *IDH1-R132C* mutation was found in 4 (2.47%) patients, *IDH2*R140Q in 3 (1.85%), *IDH1*R132H in 2 (1.23%), *DNMT3A*R882H in 2 (1.23%), and each *DNMT3A*R882C and *DNMT3A*R882P in one case (0.62%). All 13 patients (Fig. 1a) harbored a concomitant driver mutation: *JAK2*V617F, *CALR*L367fs*(type-1), or *CALR*K385fs* (type-2); one patient had a rare CALR-K360fs* mutation. We obtained from the selected patients 13 pre-transplant samples, 89 follow-up samples, 10 donor samples (donor sample was not available for 3 patients). The follow-up samples were collected at one early time point (within the first month after allo-SCT), one last-follow-up time point, and at least 3 (range 3–17) intermediate time points during the follow-up. Allele burden quantification of the MRD molecular markers was performed with digital-droplet polymerase chain reaction (ddPCR). All samples analyzed with ddPCR had a standardized DNA concentration of 24 ng/μL. The assays were carried out with QX100 Droplet Digital PCR System (Bio-Rad, Foster City, CA). DNA digestion with *Hae*III restriction enzyme was performed for *IDH1*R132C*, IDH1*R132H*, IDH2*R140Q*, JAK2* assays; with *Mse*I restriction enzyme for *DNMT3A*R882H*, DNMT3A*R882C and *DNMT3A*R882P assays*;* with *EcoR*I for *CALR* type-2 assay [8]. No DNA digestion was needed for *CALR* type-1 assay. Then, PCR mixes produced in accordance to the manufacturer protocol were transferred to the QX100 Droplet Generator (Bio-Rad), which generates approximately 20,000 droplets per well. The following protocol was used in a standard thermal cycler (Bio-Rad): denaturation (95 °C for 10 min), amplification cycles (denaturation: 94 °C for 30 s, annealing/elongation for 1 min; 40 times), a ramp rate of 1.5 °C/s, and a final 10-min inactivation step at 98 °C. We used the following annealing temperatures: 55° for *IDH1*, *IDH2*; *DNMT3A* and *JAK2* assays, 60°for *CALR* type-1 and 63° for *CALR* type-2 assay [17]. Individual wells were analyzed simultaneously for FAM(6-carboxyfluorescein) and HeX (6-carboxy-2,4,4,5,7,7-Hexachlorofluorescein succinimidyl ester) using the QX100 droplet reader (Bio-Rad). All Probes had BHQ1 quencher at the 3′ end. In order to validate the ddPCR assays, we tested them on 46 PB samples from healthy subjects. Then, we prepared progressively diluted samples at known allele frequency (5%, 1%, 0.5%, 0.1%, 0.05%, 0.01%) for each investigated mutation. We performed ddPCR assays for *IDH1*R132C, *IDH1*R132H, *IDH2*R140Q, *DNMT3A*R882H, *DNMT3A*R882C or *DNMT3A*R882P comparing progressive dilutions of each mutation with a WT-only sample (pool of healthy subject DNA), in order to determine the limit of detection (LOD) of each assay. The measured LOD was 0.05 % for *IDH1*R132C*, IDH2*R140Q*, DNMT3A*R882C *and DNMT3A*R882H mutations, 0.1% for the *IDH1*R132H *and DNMT3A*R882P mutations. Data from ddPCR assays were analyzed with QuantaSoft software (Bio-Rad). We also quantificated in the follow-up samples donor chimerism by real-time quantitative PCR using hydrolysis probes (TaqMan technology, Life Technologies, Carlsbad, CA), applying our own repertoire of qPCR assays based on a broad InDel-panel [18, 19]. The mean concentration of target sequences (copies/microliter) was calculated by the in-built Poisson algorithm. The median allele frequency of *IDH1*, *IDH2*, or *DNMT3A* at the basal time was significantly lower than the concomitant driver mutation allele frequency (median 29.80 vs 49.6%, respectively, t-Test p = 0.03). Allele frequency of IDH1/2/DNMT3A by NGS was similar to ddPCR quantification at the basal time, as shown in Fig. 1b. The results of *IDH1/IDH2/DNTMT3A* and *JAK2/CALR* concomitant quantification were concordant in 70/84 cases (83.33%). Six *JAK2*-positive and one *CALR*-positive samples were negative for *IDH1, IDH2* or *DNTMT3A*. Five *IDH1*-positive and two *IDH2*-positive samples were negative for the concomitant driver mutation. *IDH1/IDH2/DNTMT3A* and *JAK2/CALR* allele frequencies covariates with a Pearson´s ρ coefficient of the distribution is 0.944. In order to display the correlation between the two markers, eliminating the disturbing effect of different basal levels, the follow-up data for each marker were normalized according to the basal percentage of allele burden. (Fig. 1c). During the post-transplant follow-up (Fig. 1d–f), 6 patients with *IDH1* mutation and concomitant *JAK2* mutations achieved early molecular remission after allo-SCT. We observed two molecular relapses (UPN#1, UPN#12) with simultaneous positivity of both MRD ddPCR assays (*JAK2*, *IDH1*). Patient UPN#12 was successfully treated with donor lymphocyte infusions (DLI), with a recovery of long-lasting molecular remission. Two *IDH2*-mutated patients (UPN#2, UPN#5) reached early molecular remission after allo-SCT. One patient (UPN#6) failed the first allo-SCT, with rapidly progressive loss of donor chimerism and increase of JAK2 allele frequency; this patient had at the basal time point before allo-SCT a low IDH2 allele frequency (2.19%), and the *IDH2* mutation was not detectable despite the hematological persistence of the disease. The patient underwent a second allo-SCT resulting in complete molecular remission, but died after 62 days because of severe sepsis. Four *DNMT3A*-mutated patients achieved full donor chimerism in the early post allo-SCT phase: one of them (UPN#9) experienced a secondary decrease of donor chimerism (lowest 92.3% at day 91) with simultaneous increase in both *CALR* (2.02%) and *DNMT3A* (2.02%) allelic frequencies, that was resolved during the tapering of immunosuppression.

Overall, we found that molecular persistence or relapse of malignant clones was detectable by ddPCR for driver (*JAK2/CALR*), but also non-driver (*IDH1/IDH2/DNMT3A*) mutations. In particular, the results of different assays addressing driver mutations and *IDH* or *DNMT3A* are highly concordant, when normalized to the basal (pre-transplant) allele frequencies. We observed discordant results only in one case, where the *IDH2* ddPCR assay failed to detect the relapse, whereas JAK2 allele frequency was high. In this case, the pre-transplant sample showed very low levels of the *IDH2* mutation, suggesting the presence of a minimally expressed sub-clone that was not present at relapse. Our interpretation of these data is that the choice of MRD marker for each patient should take into account the pre-transplant allele frequency of different mutations. Furthermore, our results demonstrate that MRD detection by high-sensitivity ddPCR assay is feasible on peripheral-blood samples. No comparative data is available in our setting between the accuracy of MRD monitoring by ddPCR assay on peripheral blood and bone marrow samples. A better sensitivity of bone marrow MRD was demonstrated previously, e.g., in the monitoring of *NPM1*- and *PMLRARα*-positive AML by qPCR [20]. Therefore, future projects will have to include comparative studies on different samples.

We conclude that ddPCR assays for *IDH1*, *IDH2,* and *DNMT3A* mutations may be used for the molecular monitoring of triple-negative patients and may be considered in patients harboring driver mutations at low allele frequency. Our results thus confirm the hypothesis that the molecular follow-up of malignant clones in myelofibrosis is feasible and reliable based on the quantification of defined additional mutations.
